# External Outflow Graft Stenosis in HeartMate 3 Left Ventricular Assist Devices: The Dangers of Stenosis Migration

**DOI:** 10.1097/MAT.0000000000002507

**Published:** 2025-07-18

**Authors:** Parsa Jahangiri, Jeroen Wilschut, Yusuf Z. Sener, Alina C. Constantinescu, Joost Daemen, Kadir Caliskan

**Affiliations:** From the Thoraxcenter, Department of Cardiology, Cardiovascular Institute, Erasmus MC-University Medical Center, Rotterdam, the Netherlands.

**Keywords:** HeartMate 3, mechanical circulatory support, computed tomography angiography, outflow graft obstruction, pump malfunction

## Abstract

External outflow graft obstruction (eOGO) is an increasingly recognized long-term complication in patients with HeartMate 3 left ventricular assist devices. While percutaneous treatment provides a less invasive alternative to surgery, it introduces the challenge of stenosis migration—especially when graft twist is a contributing factor. This migration can extend to the edge or even beyond the Bend Relief toward the aortic or pump anastomosis, posing procedural risks in fragile anatomical zones. In this case series, we present three patients with eOGO who experienced migration of the stenotic lesion during percutaneous treatment. In the first case, the procedure was aborted due to lesion migration close to both anastomotic sites. In the second, full-graft stenting resolved the obstruction but required stenting close to the aortic anastomosis, raising concern for rupture. In the last, a preemptive “migration-aware” strategy—placing a protective stent near the anastomosis before treating the main lesion—safely contained the obstruction and prevented high-risk migration. We propose that in patients with obstruction migration or when the initial stenosis is already near an anastomotic site, clinicians should consider a migration-aware stenting strategy. This approach may help avoid uncontrolled stenosis shifts, reduce the need for high-risk anastomotic stenting, and improve procedural safety.

External outflow graft obstruction (eOGO) is an increasingly recognized complication in patients with a HeartMate 3 (HM3) left ventricular assist device (LVAD).^1–4^ The underlying mechanism often involves the accumulation of acellular debris between the outflow graft and the Bend Relief (BR) or polytetrafluoroethylene covering, with graft torsion or a combination of both also contributing in some cases.^1,5,6^ To reduce outflow graft twisting in HM3 devices, a U-shaped clip was introduced in 2018, followed by a pump design change in 2019 targeting torsion within the BR. Since then, no prominent twisting has been observed to the authors knowledge, and accumulation of acellular biodebris between the outflow graft and BR has become the primary mechanism of eOGO.^1,4^

Percutaneous treatment offers a safer alternative to surgical revision, which carries high procedural mortality.^4,7,8^ However, this approach introduces a distinct challenge: ballooning or stenting can displace the obstructive material, a phenomenon often referred to as the “toothpaste effect.”^5,9^ This displacement may result in stenosis migration, potentially to the edge or even beyond the BR.^5^ In such cases, the obstruction may shift toward the aortic or pump anastomosis, both of which are fragile and technically challenging to treat. Although such distal migration has been described once before, it remains a rarely acknowledged risk in current literature.^5^ Previous reports have proposed long self-expanding stents to address these migrations.^5^ While this technique may help cover the full-graft length, it does not prevent the formation of new stenoses near the anastomotic sites, where additional stenting could carry a high risk of rupture.

In this case series, we describe three patients with eOGO who experienced stenosis migration during percutaneous treatment. Based on these cases, we propose a “migration-aware” stenting strategy aimed at proactively protecting vulnerable anatomical regions and minimizing procedural risk. Written informed consent for data collection was given by all patients.

## Case 1

**Figure 1.**
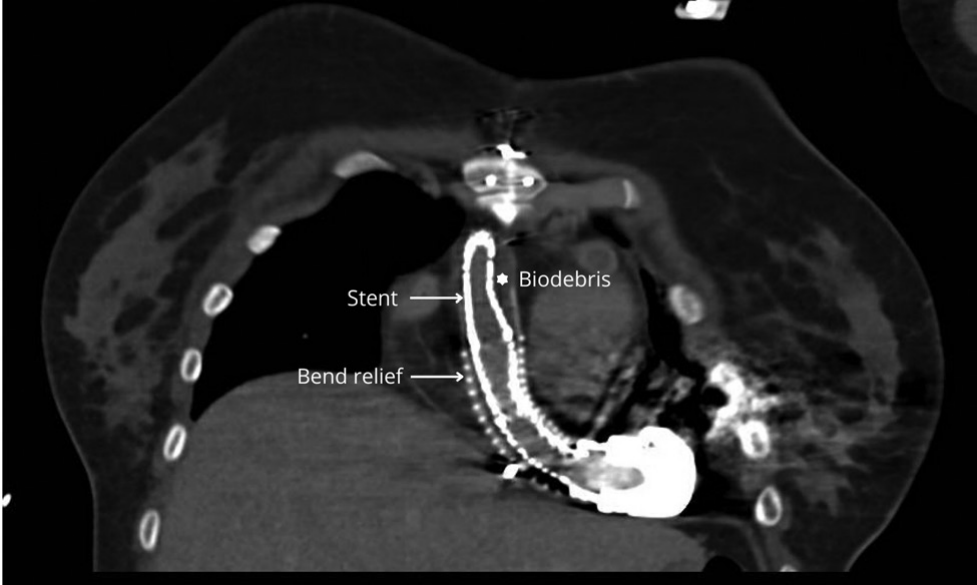
CTA scan 1 year after stenting of the OG, showing good proximal expansion of the stented segment. Mild tapering is seen at the cranial (distal) portion of the stent, near the aortic anastomosis, likely due to migrated biodebris during the initial procedure, resulting in residual external compression. CTA, computed tomography angiography; OG, outflow graft.

The first patient was a 22 year old female with asymptomatic eOGO detected 1.8 years after HM3 implantation, initially presenting asymptomatic but occasionally with low-flow alarms. Computed tomography angiography (CTA) showed progression from 40% to 50% stenosis over 3 months, and therefore, we opted for endovascular intervention. Angiography revealed a proximal stenosis near the LVAD pump with a pressure gradient of 28 mm Hg. Balloon-expandable stenting (Bentley Begraft) relieved the obstruction but immediately caused distal migration of the stenosis. During stent deployment, LVAD speed was temporarily reduced to the lowest setting (3,000 revolutions per minute [rpm]) to minimize forward flow through the outflow graft. Systemic anticoagulation with intravenous heparin was administered throughout the procedure. This approach was applied consistently in all subsequent cases. A second stent was deployed, leading to repeated migration both proximally and distally (Supplementary Video). Notably, angiography suggested that the new distal stenosis was located beyond the BR, near the aortic anastomosis, while the proximal migration approached the pump housing. Because both new sites lay near surgically delicate anastomoses, and due to concern over rupture risk, further stenting was withheld. The final pressure gradient was 23 mm Hg. Despite limited improvement, the result was accepted given the absence of symptoms and the risk associated with further manipulation. A CTA scan performed 1 year post-stenting showed good proximal stent expansion, with mild tapering distally near the aortic anastomosis, corresponding to the site of the obstruction previously seen on angiography, likely due to migrated biodebris causing residual external compression (Figure 1).

## Case 2

**Figure 2.**
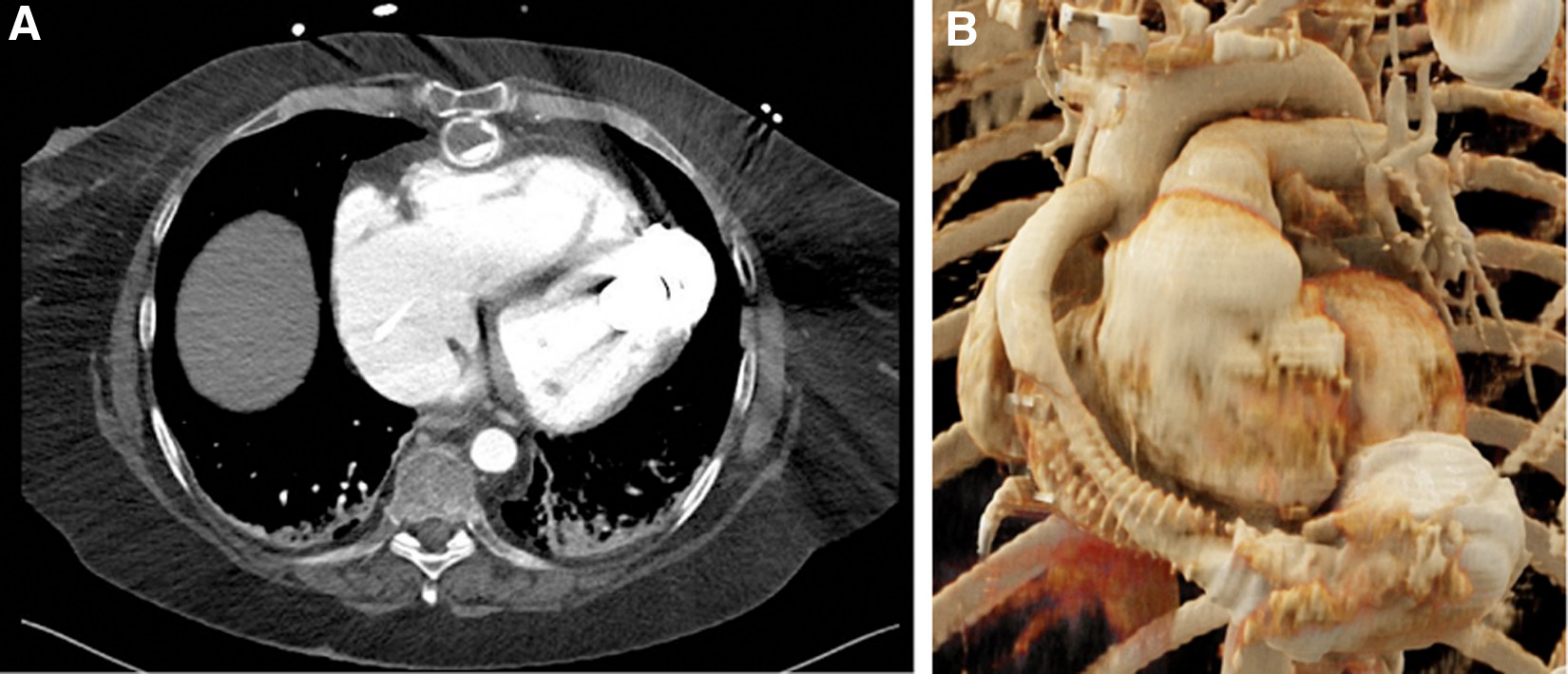
Imaging demonstrating proximal outflow graft obstruction in a 63 year old female with cardiogenic shock. **A**: Computed tomography angiography revealing a filling defect of uncertain etiology in the proximal outflow graft at the level of the Bend Relief. **B**: Three-dimensional reconstructions with the appearance of mechanical obstruction secondary to compression external to the graft. Graft torsion and/or thrombosis could not be excluded on these images.

**Figure 3.**
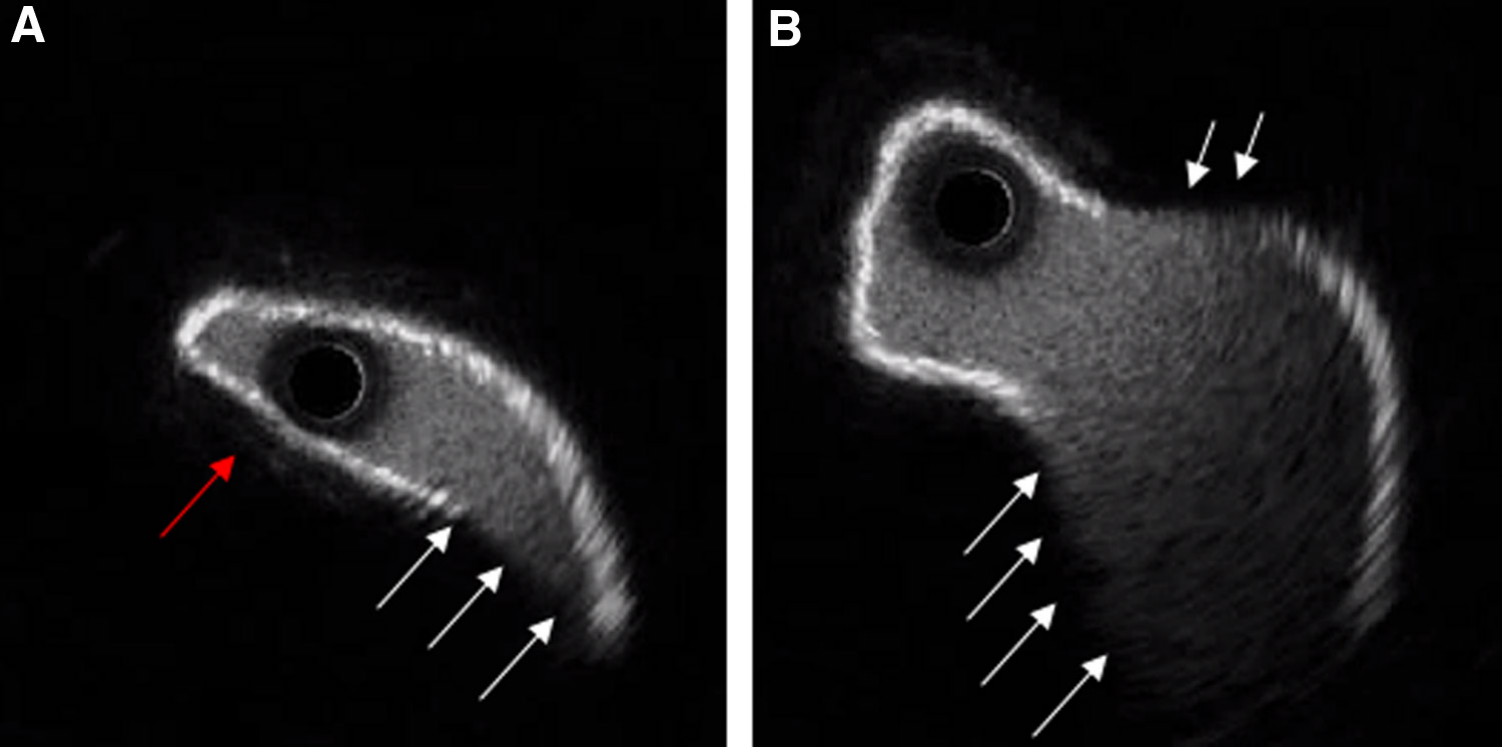
IVUS assessment of the outflow graft. **A** and **B**: IVUS before stenting demonstrates an oval-shaped luminal narrowing caused mainly by external compression (red arrow), but also some degree of torsion as indicated by the echolucent luminal borders (white arrows) as a result of twisting in the acoustic shadow of luminal borders that are in closer proximity to the IVUS catheter. IVUS, intravascular ultrasound.

In a second case, a 63 year old female presented in cardiogenic shock 29 months post-LVAD implantation. Computed tomography angiography revealed a significant filling defect in the proximal outflow graft at the level of the BR (Figure 2A). Because of the patient’s hemodynamic instability, an endovascular approach was chosen with emergency. Three-dimensional CTA suggested external compression as the likely cause; graft torsion could not be excluded on this image (Figure 2B). Hemodynamic assessment confirmed a pressure gradient of 57 mm Hg. Intravascular ultrasound (IVUS) revealed an oval-shaped narrowed lumen consistent with external compression and also signs of mild torsion (Figure 3, A and B).

**Figure 4.**
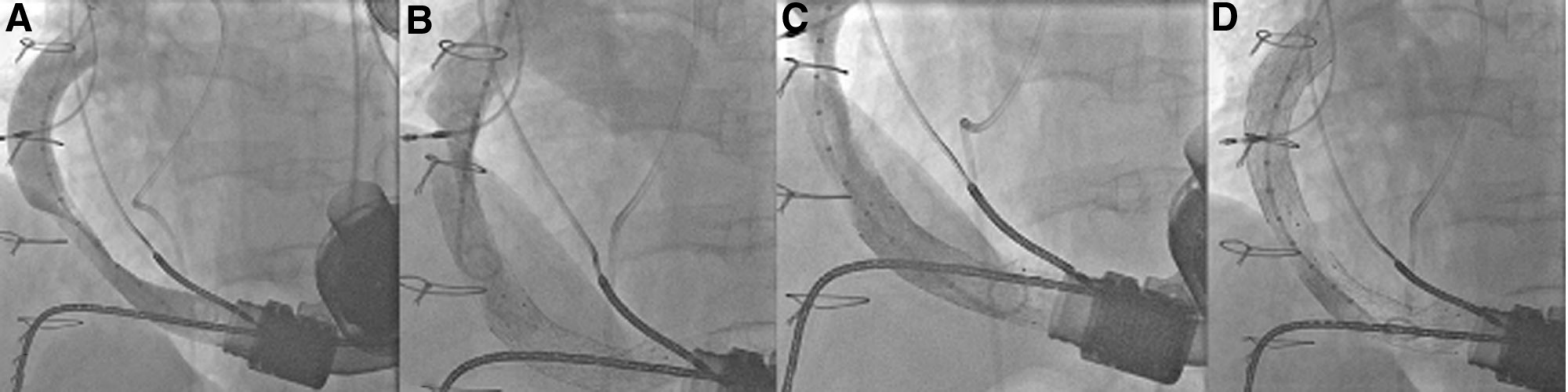
Fluoro-angiography before stenting (**A**), after placement of stent 1 demonstrating a distal shift of the stenotic segment (**B**), after placement of stent 2 demonstrating residual stenosis distally (**C**), and after placement of stent 3 (**D**).

A 14 × 60 mm self-expanding Venovo stent was placed across the lesion but caused distal migration. A second stent (14 × 60 mm Prestige) was deployed with minimal overlap, again followed by migration toward the aortic anastomosis. A third Venovo stent (14 × 60 mm) was required to cover the final stenosis (Figure 4). Although the final stenosis which needed the third stent exhibited a relatively low gradient (~10 mm Hg), we recognized that it was located dangerously close to the aortic anastomosis within a potential twisted segment, substantially heightening the risk of rupture. Fortunately, no rupture had occurred. This case further underscored how distal migration of the obstruction can lead to procedural dilemmas in high-risk anatomical zones.

## Case 3

**Figure 5.**
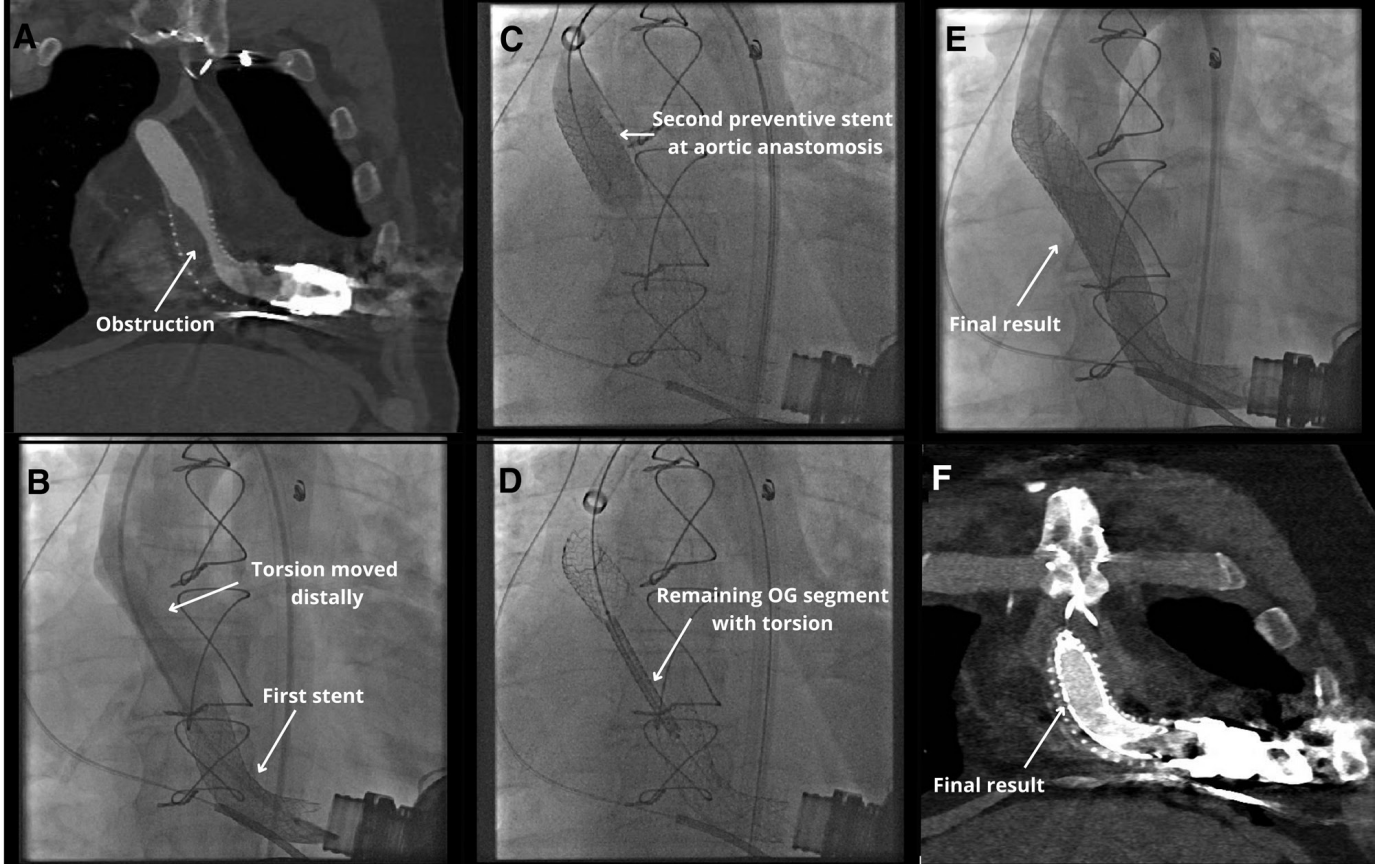
A 64 year old man with a HeartMate 3 left ventricular assist device implanted for ischemic heart disease 2 years earlier was admitted with significant stenosis on routine CTA follow-up. CTA confirmed a 65% obstruction of the outflow graft (**A**), which was validated by angiography showing a peak-to-peak pressure gradient of 28 mm Hg. This led to the placement of two balloon-expandable covered stents. However, a new stenosis developed in the distal outflow graft, with a gradient of 15 mm Hg (**B**). To prevent further expansion toward the aortic anastomosis during subsequent treatment of the new stenosis site, a prophylactic stent was first placed strategically just proximal to the aortic anastomosis (**C**). This was intended to enable safer treatment of the mid-graft obstruction (**D**). The final angiography confirmed the successful stent placement in the mid-graft (**E**) (white arrow), with a good procedural outcome, further verified by CTA (**F**). CTA, computed tomography angiography.

In the third patient, a migration-aware strategy was applied to proactively prevent complications. We propose that when there is reason to suspect graft torsion as the underlying mechanism for stenosis migration, or when the obstruction lies near an anastomotic site, clinicians should consider placing a protective stent slightly overlapping the anastomosis before proceeding with the rest of the intervention. We applied this approach in this patient. After two balloon-expandable stents (16 × 38 mm Begraft) were placed to treat the initial obstruction, the stenosis migrated distally to the end of the mid-graft. To prevent further migration toward the aortic anastomosis during subsequent interventions, a prophylactic stent (18 × 38 mm Begraft) was deployed just proximal to the anastomosis. The migrated mid-graft segment was then treated with a longer stent (20 × 48 mm Begraft), resulting in complete resolution of the obstruction and normalization of the pressure gradient (Figure 5).

## Discussion

External outflow graft obstruction is an increasingly recognized complication in patients with HM3 LVADs.^1–4^ Patients often present with low-flow alarms and signs of left ventricular failure, although asymptomatic cases are not uncommon.^1,4,10,11^ The underlying pathophysiology most commonly involves the accumulation of acellular biodebris between the outflow graft and the BR, occasionally accompanied by graft twist.^1,5^ Although prominent twist-related eOGO is no longer observed since the introduction of anti-twist mechanisms in newer HeartMate 3 models, this mechanism may still be relevant in patients implanted before the revision of the OG-to-LVAD connection, given the long-term nature of this complication. Our experience shows that this mechanism could still potentially remain a relevant finding in daily practice. Differentiating between external compression and twist remains challenging. The combination of CTA with three-dimensional reconstruction and IVUS can help clarify the underlying mechanism. Graftoplasty has also been described as a useful adjunct in complex cases.^9^

Although percutaneous treatment provides a less invasive alternative to surgical revision—which carries high procedural mortality—it introduces its own risks.^7,12^ Stenting can displace obstructive material and lead to migration of the stenosis to the edge or even beyond the BR.^5^ This can result in obstruction near the aortic or pump anastomosis—regions that are technically difficult and anatomically fragile, increasing the risk of rupture during further intervention.

Our cases highlight that migration to the edge or even beyond the BR is a clinically significant and under-recognized complication of percutaneous eOGO treatment. Although some groups have proposed full-graft coverage using long self-expanding stents, this approach does not address the risk of newly formed stenoses near anastomotic sites.^5^ If such lesions develop in high-pressure segments near these fragile regions, additional stenting could provoke rupture—even when technically feasible.

A migration-aware strategy, as demonstrated in our final case, offers a promising solution. By placing a protective stent overlapping the anastomosis before addressing the primary lesion, further migration can be contained. This approach allows safer management of complex obstructions, especially in cases where twist is suspected as a contributing mechanism.

## Conclusions

External outflow graft obstruction due to biodebris accumulation remains a common finding in HM3 patients. While percutaneous treatment can relieve the obstruction, it may also cause displacement of the obstructive material, leading to a new site of stenosis. When migration causes the stenosis to extend to the edge or even beyond the BR—particularly toward the aortic or pump anastomosis—it creates procedural challenges that demand thoughtful planning. A migration-aware strategy, involving preemptive stenting to protect the anastomotic zones, offers a safe and pragmatic approach to reduce the risk of high-risk stenting and potential graft rupture during percutaneous management of eOGO.
